# Novel 1 L polyethylene glycol-based bowel preparation (NER1006): proof of concept assessment versus standard 2 L polyethylene glycol with ascorbate – a randomized, parallel group, phase 2, colonoscopist-blinded trial

**DOI:** 10.1186/s12876-019-0988-y

**Published:** 2019-05-30

**Authors:** Lucy B. Clayton, Bola Tayo, Marc Halphen, Rüdiger Kornberger

**Affiliations:** 1 Clinical Development, Norgine Ltd, Norgine House, Moorhall Road, Harefield, Uxbridge, UB9 6NS UK; 2 GW Pharmaceuticals plc, Sovereign House, Vision Park, Chivers Way, Histon, Cambridge, CB24 9BZ UK; 3 PAREXEL International GmbH, Spandauer Damm 130, 14050 Berlin, Germany

**Keywords:** Bowel cleansing, Colonoscopy, Low-volume, Polyethylene glycol, Phase 2 clinical trial

## Abstract

**Background:**

Colonoscopy requires colon cleansing. For this, many polyethylene glycol (PEG)-based preparations still require a high preparation-volume intake. Using an increased osmotic load with ascorbate (Asc), five new low-volume PEG-based bowel preparations (LVPEG) were tested for clinical proof of concept.

**Methods:**

This two-part, open-label study examined preparation-volumes of 1–1.25 L and total required fluid volumes of 2–3 L. Part 1, in healthy volunteers, used mean cumulative 24-h stool weight (target > 2750 g) to identify a lead candidate. Part 2 was endoscopist-blinded: patients undergoing screening colonoscopy were randomized before treatment with the selected lead, one of two variants of it, or the control 2 L PEG + Asc. Two primary endpoints were used for proof of concept demonstration: mean 24-h stool weight and bowel cleansing success (Harefield Cleansing Scale).

**Results:**

A total of 120 subjects (30 per group) were enrolled/randomized 1:1:1:1 (max 40:60 gender ratio) per completed Part. In Part 1, LVPEG-3 achieved the largest mean stool weight (3399 g: *P* < 0.0001 vs target) and was selected for Part 2. In Part 2, stool weights exceeded the target, notably for LVPEG-4 (3215 g: *P* < 0.001), which achieved 100% cleansing success after a total required fluid intake of 2 L. The control achieved 90% cleansing success. Adverse events were few, gastrointestinal in nature and similar between groups.

**Conclusions:**

LVPEG-4 achieved a clinically useful combination of cleansing, safety/tolerability and low consumption volume: 1 L preparation + 1 L required additional fluid. Named NER1006, LVPEG-4 demonstrated clinical proof of concept and warrants further investigation.

**Trial registration:**

**October 2012.**

Identifier: NCT01714466. EudraCT: 2012–003052-37

The trial was prospectively registered.

**Electronic supplementary material:**

The online version of this article (10.1186/s12876-019-0988-y) contains supplementary material, which is available to authorized users.

## Background

Bowel preparation is a critical factor in the diagnostic and therapeutic success of colonoscopy and colorectal cancer (CRC) screening.

Bowel preparations based on polyethylene glycol 3350 (PEG3350) plus electrolyte solutions are well established. Ascorbic acid/sodium ascorbate (Asc), that is vitamin C, adds to the laxative effect of PEG3350 whilst also enabling a total volume reduction [1–3]. The development of a 2 L PEG + Asc preparation halved the required preparation-volume intake versus older 4 L PEGs and thus represented a more convenient but still effective bowel preparation [4–6]. As it was easier to complete, patients were more likely to be willing to undergo the procedure again if they received the 2 L PEG + Asc [4, 7, 8]. However, even with 2 L PEG + Asc, a total-fluid-volume of 3 L should be ingested in a short time, and taking 2 L of a bowel preparation is still a challenge for some patients. Further volume reductions may thus help even more patients to successfully complete their bowel-preparations.

It was hypothesized that an effective low-volume bowel preparation could be achieved with an increased ascorbate amount/osmotic load. An initial clinical study exploring new low-volume PEG + Asc (LVPEG) split-dosing bowel preparations identified two test formulations suitable for further clinical investigation [data on file: Norgine]. The current study used taste- and flavor-optimized versions of these formulations to assess if they could repeat stool output achievements and demonstrate clinical proof of concept by achieving functional bowel cleansing.

## Methods

### Trial design

This was a two-part, randomized, Phase 2 study, conducted at the PAREXEL clinical trial unit in Berlin, Germany (for registration/protocol see: ClinicalTrials.gov NCT01714466; EudraCT 2012–003052-37). Part 1 of the study was open-label and conducted in healthy volunteers who did not require a colonoscopy. It aimed to assess the pharmacodynamics (stool output); and to establish a suitable administration sequence for the two doses, the reconstitution volume and the required additional fluid volume. Proof of concept was assessed in Part 2: in patients undergoing a screening colonoscopy. This Part was endoscopist-blinded (the endoscopist was unaware of the preparation administered) and used a validated bowel cleansing scale to assess cleansing quality. Safety and tolerability were assessed in both study Parts.

The study was approved by the Local Independent Ethics Committee and performed according to the principles of the Declaration of Helsinki, the Guidelines of the International Conference on Harmonization on Good Clinical Practice, and the European Union Clinical Trials Directive [9–11].

### Eligibility

In Part 1, healthy male and female subjects aged 40 to 70 years were enrolled. Subjects were ineligible if they had a history of clinically significant gastrointestinal (GI) symptoms or current acute abdominal discomfort or symptoms. In Part 2, enrolled patients were scheduled to undergo a screening colonoscopy and they were either 40 to 70 years old with a known personal or familial risk of contracting CRC, or 55 to 70 years old. Women of child-bearing potential were required to use adequate contraception. Full exclusion criteria are provided in Additional file 1: Table S1. In summary, the main exclusion criteria were: presence of current, clinically significant, functional GI disorder (e.g. gastric emptying disorder, chronic constipation, irritable bowel syndrome); regular use of laxatives or colon motility altering drugs in the last month before study drug administration; any history or current presence of ileus, GI obstruction or perforation, GI tract cancer, inflammatory bowel disease, or colonic resection; and history or evidence of any clinically significant cardiovascular or neurological disease, or cardiac, renal, or hepatic insufficiency. Subjects were also ineligible if they had any clinically relevant physical findings or deviations of laboratory parameters. For all subjects, ferrous sulfate was stopped at least 1 week prior to study medication.

### Randomization and treatment

All patients in both parts of the study were enrolled consecutively. In Part 1, subjects were randomized (1:1:1:1) and stratified by gender, with blocks of four receiving any one of three novel LVPEGs (LVPEG-1, LVPEG-2, LVPEG-3) or the control preparation: 2L PEG + Asc (MOVIPREP®) (Table 1). A 50:50 gender ratio was targeted with a randomization list per gender and a maximum ratio of 60:40 was allowed. The randomization was created using the PROC PLAN procedure in SAS®. A randomization number, corresponding to a treatment pack, was allocated to each eligible subject. The control was selected based on its convenience versus standard 4L PEGs and its wide use as a first line treatment. In Part 2, patients were similarly randomized, receiving any out of LVPEG-3, one of two LVPEG-3 variants (LVPEG-4 or LVPEG-5), or the control (Table 1).

**Table 1:** Studied Bowel Preparations. Composition and Required Fluid Volumes of the LVPEGs and Control used in this Study^a^

Dosing Regimen		LVPEG-1		LVPEG-2		LVPEG-3		LVPEG-4		LVPEG-5		Control	
Study Part		1		1		1 and 2		2		2		1 and 2	
Dose		Evening	Morning	Evening	Morning	Evening	Morning	Evening	Morning	Evening	Morning	Evening	Morning
Fluid Volume (mL)													
	Preparation	750	500	500	750	500	500	500	500	500	500	1000	1000
	Preparation, Total	1250		1250		1000		1000		1000		2000	
	Required Additional Fluid	875	875	875	875	1000	1000	500	500	1000	1000	500	500
	Required Additional Fluid, Total	1750		1750		2000		1000		2000		1000	
	Required Fluid, Total	3000		3000		3000		2000		3000		3000	
Dose Composition (g)													
	PEG3350	100	40	40	100	100	40	100	40	100	40	100	100
	Sodium Sulfate	9	-	-	9	9	-	9	-	9	-	7.5	7.5
	Sodium Ascorbate	-	48.1	48.1	-	-	48.1	-	48.1	-	40	5.9	5.9
	Ascorbic Acid	-	7.5	7.5	-	-	7.5	-	7.5	-	-	4.7	4.7

^a^Osmotically active ingredients only. All formulations included balanced electrolytes sodium chloride and potassium chloride

Total Required Fluid Volume = Bowel Preparation Reconstituted Volume + Mandatory Additional Clear Fluid Volume

### Test formulations and colonoscopy

Each LVPEG consisted of two different powders for reconstitution into oral solution for split evening and morning dosing. To produce the cathartic effect, LVPEGs had PEG3350, Asc and sodium sulfate in varying amounts across the two doses. They also contained potassium chloride and sodium chloride, which were used to replace electrolytes lost through the osmotic effects of the formulations. The control had the same amounts of PEG, Asc and sodium sulfate in each dose. Compared to the control, LVPEGs contained less PEG and sodium sulfate, but about 2.5× the amount of Asc (except for LVPEG-5, which contained about 2× the amount of Asc). Each formulation was also taste- and flavor-optimized.

LVPEG-1 and LVPEG-3 both included Asc in the morning dose, whereas LVPEG-2 (LVPEG-1 regimen in reverse order) was the only LVPEG to have Asc in the evening dose. Preparation reconstitution volumes varied from 500 to 750 mL per dose. Based on its low preparation volume, significant effect on 24-h stool weight, and acceptable tolerability, LVPEG-3 was selected as the lead candidate for Part 2 of the study. Colonoscopy patients thus received LVPEG-3; one of its two variants, LVPEG-4 (with a lower required-additional-fluid-volume) or LVPEG-5 (with less Asc); or the control.

In both Parts 1 and 2 of the study, all bowel preparations were administered in an overnight split-dosing fashion following a standardized meal plan where all participants ate the same breakfast (09:00), snack (11:00) and light lunch (14:00). There were no food restrictions specified in the period before the study began. The first (evening) dose was taken on Day 1 of the study, after fasting from 14:00. Between 17:00 and 18:00, intake began over a maximum of 2 h. On Day 2, after overnight fasting and starting between 07:00 and 08:00, the second (morning) dose was taken over a maximum of 2 h. After each dose, variable volumes of mandatory additional clear fluid were needed to make up a total mandatory fluid volume of 3 L (or 2 L for LVPEG-4). Subjects could also consume ad libitum fluid as their thirst dictated; these volumes were recorded in Part 2 and included in the total fluid volumes.

The first meal was provided 4 h after completion of the second dose, after the safety laboratory blood test and (in Part 2) the colonoscopy.

### Primary endpoints

Part 1 of the study assessed the pharmacodynamic endpoint of stool weight output >2750 g over 24 h from the start of the evening dose. This value was based on Phase 1 study findings [data on file: Norgine] and previous observations on stool weight output and correlations to colon cleansing in healthy volunteers [12, 13].

Part 2 had two primary endpoints, both exploratory: stool weight output over 24 h and overall bowel cleansing success rate according to the Harefield Cleansing Scale (HCS) [14].

Stool weight was determined for each collected stool fraction. For the HCS, grades A and B were classified as successful, meaning that the colon was empty, clean and with the mucosa fully visualized or that full visualization could be achieved with appropriate washing/suctioning. Grades C and D were classified as unsuccessful.

### Secondary endpoints

Secondary endpoints in both parts of the study included tolerability, as assessed by vomiting rates, and the time and fluid volume required to reach clear effluent within 24 h, as assessed by the study investigator. The clear effluent is a surrogate marker of bowel cleansing, defined here as stool appearance being both ‘clear’ and ‘liquid’. Additional endpoints in Part 2 only were the segmental colon cleansing scores using the HCS and pharmacokinetic (PK) assessment of active ingredients in feces, plasma and urine to assess biological activities. These samples were collected 0–360 min after the consumption of each dose.

### Statistics

The full analysis set (FAS) included all subjects who met major entry criteria, had received study medication at least once and for whom post-baseline data for the primary endpoint were available. The safety population (SAF) included all subjects who received any amount of study medication. All safety analyses were based on the SAF. The PK population in Part 2 included participants who consumed at least 75% of each dose and who had at least the parameters C_max_ and AUC_0-t_ evaluable.

In Part 1, a sample size of 25 subjects per group was required to enable demonstration of a mean stool output in excess of 2750 g. This was given an effect size of 0.5 and a power of 80% when the expected mean ± standard deviation (SD) value was 3000 ± 500 g. To allow for dropouts or non-evaluable cases, 30 subjects per group were enrolled to provide at least 25 fully evaluable subjects per group. A parametric result analysis was performed, using a one-sample t-test by dosing regimen.

In Part 2, the sample size was arbitrarily set at 30 patients per treatment group to provide useful information for a qualitative proof of concept. Potential cleansing success rate differences between LVPEGs and the control were assessed with a pair-wise Fisher’s exact test.

Safety data were listed and summarized.

## Results

### Patient disposition and characteristics

**Fig. 1:**
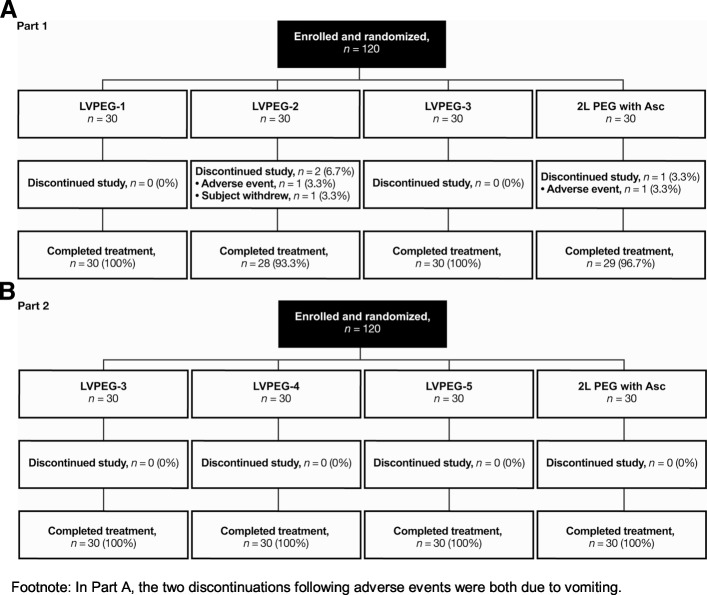
Subject Disposition. Consort Diagrams for **a**) Part 1, and **b**) Part 2 of the Study

The study was conducted between 22nd October 2012 and 8th July 2013. Out of 120 enrolled participants 117 completed Part 1 of the study (Fig. 1a). In Part 2, all 120 enrolled participants completed the study (Fig. 1b). Baseline characteristics (Table 2) were comparable between treatment groups. All 120 subjects enrolled in Part 1 were included in the safety population. Two subjects discontinued the study after vomiting after part (LVPEG-2) or all (control) of the first dose. All 120 patients enrolled in Part 2 were included in the safety and FAS populations. One patient was excluded from the PK population as they were given the two doses in the wrong order by mistake.

**Table 2:** Baseline Characteristics

	LVPEG-1	LVPEG-2	LVPEG-3	Control	LVPEG-3	LVPEG-4	LVPEG-5	Control
Study Part	1	1	1	1	2	2	2	2
Patients, n	30	30	30	30	30	30	30	30
Age, years (mean ± SD)	53.2 ± 8.2	53.4 ± 8.0	55.4 ± 8.2	51.2 ± 8.4	60.4 ± 5.7	60.0 ± 6.3	61.9 ± 6.7	58.8 ± 6.1
Gender, n (%)								
Male	17 (56.7)	17 (56.7)	18 (60.0)	18 (60.0)	14 (46.7)	13 (43.3)	13 (43.3)	14 (46.7)
Female	13 (43.3)	13 (43.3)	12 (40.0)	12 (40.0)	16 (53.3)	17 (56.7)	17 (56.7)	16 (53.3)
Race, n (%)								
White or Caucasian	30 (100.0)	29 (96.7)	30 (100.0)	30 (100.0)	30 (100.0)	30 (100.0)^a^	30 (100.0)	30 (100.0)
Other	0	1 (3.3)	0	0	0	0	0	0
BMI (kg/m^2^, mean ± SD)	25.4 (2.8)	25.5 (2.2)	25.2 (2.8)	25.0 (3.3)	25.4 (2.7)	25.9 (3.4)	25.8 (2.9)	25.4 (3.4)

^a^One subject, receiving LVPEG-4 in Part 2, was described as both Hispanic and White/Caucasian. SD, standard deviation; BMI, body mass index

### Stool weight output

**Fig. 2:**
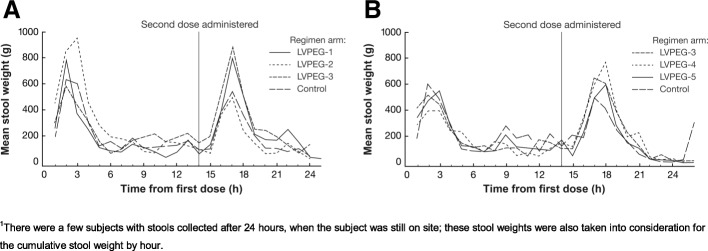
Stool Weight Output. Mean Hourly Stool Weight Output (g) from Start of Dosing for 24 Hours ^1^ in **a**) Part 1, and **b**) Part 2 of the Study

LVPEGs and the control all reliably triggered stool weight outputs in both Part 1 (Fig. 2a) and Part 2 (Fig. 2b) of the study, with peak output levels around 2–3 h after each dose.

**Fig. 3:**
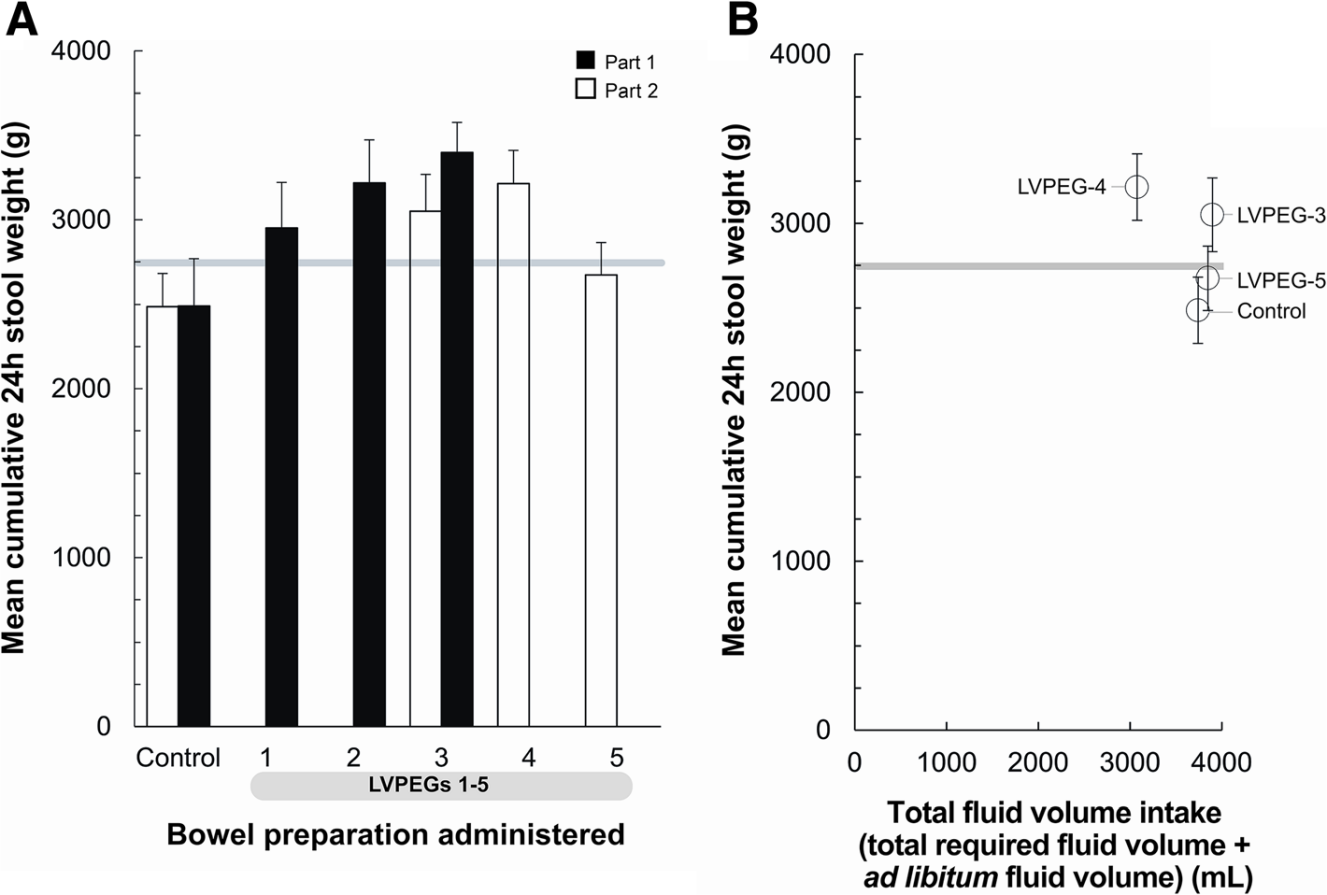
Mean Cumulative 24-h Total Stool Weights. Mean Stool Weights (g) with 90% CI (g). **a**) Summary of Mean 24-h Stool Weights. Black bars indicate Part 1 of the study; white bars indicate Part 2. **b**) Mean 24-h Stool Weights vs Total Fluid Volume (Total Required Fluid Volume + Ad Libitum Fluid Volume) from Part 2 of the study. Volumes in mL. The horizontal grey line indicates the 2750 g mean stool weight target that was to be exceeded

In Part 1, a mean cumulative 24-h stool weight significantly above the 2750 g target was achieved by LVPEG-2 (3219 g, *P* = 0.0042) and LVPEG-3 (3399 g, *P* < 0.0001), but not by LVPEG-1 (2951 g, *P* = 0.2176) or the control (2491 g, *P* = 0.8764) (Additional file 2: Table S2, Fig. 3a). The target was also exceeded numerically by all LVPEGs (but not the control) regardless of the preparation volume (1–1.25 L; Additional file 3: Figure S1A) or total required fluid volume (3 L; Additional file 3: Figure S1B). Since LVPEG-3 achieved the highest mean stool weight, and was well tolerated, it was selected as the lead for Part 2.

In Part 2, LVPEG-4 achieved a mean stool weight comparable to that of LVPEG-3 (Additional file 2: Table S2, Fig. 3a), with a preparation volume of 1 L (Additional file 3: Figure S1A), a total required fluid volume of 2 L (Additional file 3: Figure S1B) and a consumed mean total fluid volume of only 3077 mL (Fig. 3b). All ingested mean total fluid volumes (including ad libitum) exceeded 3 L (3077–3897 mL). The 2750 g target mean stool weight was exceeded by LVPEG-3 (3050 g, *P* = 0.0268) and LVPEG-4 (3215 g, *P* = 0.0004), but not by LVPEG-5 (2675 g, *P* = 0.4907) or the control (2487 g, *P* = 0.9691).

LVPEG-3 and the control both achieved similar stable stool output levels across both parts of the study.

### Clinical bowel cleansing efficacy

**Fig. 4:**
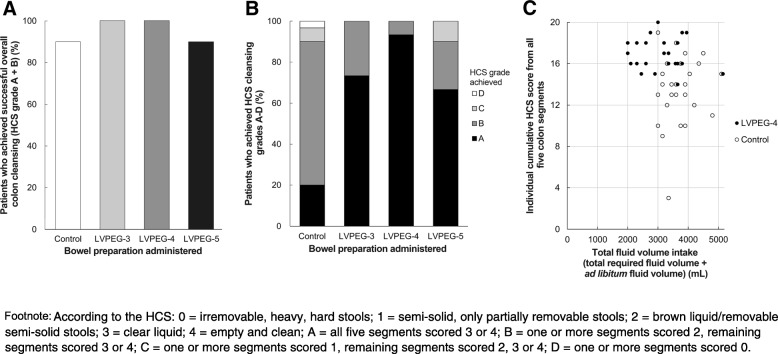
Overall Colon Cleansing Efficacy using the HCS (Part 2). **a**) Proportion of Patients who Achieved Successful Overall Colon Cleansing (HCS A + B). **b**) Proportion of Patients who Achieved HCS Cleansing Grades A–D. **c**) Individual cumulative HCS Scores from all Five Colon Segments vs Total Fluid Volume (Total Required Fluid Volume + Ad Libitum Fluid Volume) Intake (mL). Data showing LVPEG-4 vs Control to demonstrate that clinically useful cleansing is possible with a low total volume intake, even if many patients voluntarily preferred to drink more than 3 L in total

LVPEG-3 and its two variants, LVPEG-4 and LVPEG-5, were clinically assessed in patients undergoing a colonoscopy procedure in order to determine a proof of concept between the recorded stool output levels and bowel cleansing efficacy seen during colonoscopy. The overall bowel cleansing success rates were [% subjects (90% CI; P vs control)]: LVPEG-3: 100% (0.88–1.00; *P* = 0.237), LVPEG-4: 100% (0.88–1.00; P = 0.237), LVPEG-5: 90% (0.02–0.27; *P* = 1.000), and 90% for the control (Fig. 4a). Grade B on the HCS represents the threshold for cleansing success; it disqualifies segmental scores below 2. With mean segmental cleansing scores exceeding 2.8, LVPEG-3, LVPEG-4, and LVPEG-5 demonstrated clinically useful cleansing efficacy in all colon segments, as did the control regimen with mean scores 2.6–3.0 (Additional file 4: Table S3). Unlike its comparators, LVPEG-4 achieved over 90% high-quality (HCS grade A) cleansing (Fig. 4b). A total of 29/30 patients on LVPEG-4 reached individual cumulative segmental HCS scores of 15 or higher out of a maximum of 20, regardless of their total fluid volume intake (Fig. 4c). At an average of 3004 ± 718 mL (SD) for the LVPEG-4 group the total fluid volume intake was lower than for the control group (3667 ± 530 mL; *P* < 0.001).

### Reaching clear effluent

In Part 1 of the study the responder rate for reaching clear effluent within 24 h was 63.3% for LVPEG-1, 58.6% for LVPEG-2, and 90.0% for LVPEG-3. In the control group 46.7% of subjects reached clear effluent. In Part 2 of the study clear effluent was achieved by 83.3% of subjects on LVPEG-3, 80% of subjects on LVPEG-4 and 76.7% of subjects on LVPEG-5. In the control group 63.3% reached clear effluent, a slightly higher proportion than in Part 1. LVPEG-3 and LVPEG-4 showed the highest responder rates, with the advantage of LVPEG-4 being that it required a total fluid consumption of only 2 L versus 3 L required for LVPEG-3.

### Treatment compliance

Consumption of < 75% of either dose was defined as protocol deviation. In both study parts compliance levels were high. In Part 1 it was 100.0, 97.6, 100.0 and 97.8% for LVPEG-1, LVPEG-2, LVPEG-3 and the control, respectively. In Part 2 it was 100.0% for LVPEG-3, LVPEG-5 and the control, and 99.8% for LVPEG-4.

### Tolerability: vomiting (patient-reported rates)

Vomiting affected between 3.33–6.9% and 0–3.3% of subjects in each group in Parts 1 and 2 respectively (Table 3). The overall number of subjects who vomited in each group in Part 2 was [n (P vs control)]: LVPEG-3: 1/30 (*P* = 1.000); LVPEG-4: 1/30 (*P* = 1.000); LVPEG-5: 0/30 (not applicable), and 0/30 for the control.

**Table 3:** Safety/Tolerability

	LVPEG-1	LVPEG-2	LVPEG-3	Control	LVPEG-3	LVPEG-4	LVPEG-5	Control
Study Part	1	1	1	1	2	2	2	2
Patients, n	30	30	30	30	30	30	30	30
Subjects with TEAEs^a^, n (%)	17 (56.7)	14 (46.7)	19 (63.3)	15 (50.0)	18 (60.0)	15 (50.0)	16 (53.3)	11 (36.7)
Subjects with TEAEs Leading to Withdrawal, n (%)	0	1 (3.3)	0	2 (6.67)	0	0	0	0
Number of Related TEAEs	29	24	35	28	24	25	24	15
Patients with Specific TEAEs^b^, n (%)								
Abdominal Discomfort	1 (3.3)	1 (3.3)	3 (10.0)	3 (10.0)	2 (6.7)	3 (10.0)	4 (13.3)	1 (3.3)
Abdominal Distension	0	0	2 (6.7)	4 (13.3)	0	2 (6.7)	1 (3.3)	1 (3.3)
Abdominal Pain	6 (20.0)	2 (6.7)	0	4 (13.3)	3 (10.0)	1 (3.3)	3 (10.0)	3 (10.0)
Nausea	6 (20.0)	8 (26.7)	10 (33.3)	7 (23.3)	6 (20.0)	5 (16.7)	3 (10.0)	3 (10.0)
Vomiting	1 (3.3)	3 (10.0)^c^	1 (3.3)	1 (3.3)	1 (3.3)	1 (3.3)	0	0
Fatigue	0	0	1 (3.3)	0	2 (6.7)	0	1 (3.3)	1 (3.3)
Malaise	0	0	3 (10.0)	0	1 (3.3)	4 (13.3)	2 (6.7)	2 (6.7)
Thirst	0	0	1 (3.3)	0	2 (6.7)	0	0	0
Dizziness	2 (6.7)	1 (3.3)	0	0	1 (3.3)	3 (10.0)	1 (3.3)	0
Headache	7 (23.3)	4 (13.3)	6 (20.0)	4 (13.3)	5 (16.7)	3 (10.0)	3 (10.0)	6 (20.0)
Skin Irritation	0	2 (6.7)	2 (6.7)	0	0	0	0	0

TEAEs, treatment-emergent adverse events; MedDRA, Medical Dictionary for Regulatory Activities. ^a^There were no serious TEAEs or TEAEs leading to death. ^b^Reported with a frequency ≥ 5%. ^c^One subject was excluded from the vomiting rate analysis due to a protocol violation (compliance < 75%)

### Pharmacokinetics

Across the two doses most of the PEG was eliminated within 24 h. Plasma AUC_0-t_ values showed comparable total systemic exposure to ascorbic acid for LVPEGs and the control group. Absorption of ascorbate components, as a proportion of the dose contained in the LVPEGs, was low. Plasma levels of oxalic acid, a metabolite of ascorbic acid, were below the detectable limit. Urine and fecal PK parameters indicated that osmotically active components were minimally absorbed in the intestine, thus contributing to the osmotic load, and excreted largely through the colon (Additional file 5: Table S4, Additional file 6: Table S5 and Additional file 7: Table S6). In both parts of the study most subjects had plasma electrolytes (chloride, potassium, and sodium) within the normal range at baseline and after treatment administration. A shift from normal to high was observed in a small proportion of patients. Most shifts were observed in the control group in Part 1 where 13.3% (*n* = 4) of subjects had elevated chloride values on Day 2, and in the LVPEG-4 treatment group in Part 2 where 13.3% (n = 4) of patients had elevated potassium and chloride values, and 16.7% (*n* = 5) of subjects had elevated sodium values. None of these shifts were considered clinically relevant.

### Safety

A summary of treatment-emergent adverse events (TEAEs) is presented in Table 3. The incidence of TEAEs was similar among treatment groups for both study parts. Gastrointestinal events were the most common types of TEAEs. Most TEAEs were assessed as related to the investigational product and of mild intensity. There was one TEAE of severe intensity (pain in extremity, Part 2, LVPEG-5), which was assessed as unrelated to study medication. There were no serious adverse events (AEs) or deaths during the study, and no other significant AEs.

### Tolerability questionnaire

Tolerability was based on a subject questionnaire with 3 questions per dose and 2 questions after completion of both doses. In Part 1, per evening/morning intake, most subjects rated tolerance as ‘good’ or ‘acceptable’ and occurrence of symptoms as ‘none’. The taste of the solution was rated 31.1–44.2 on a visual analog scale of 0–100. Overall, most subjects rated ease of drinking as ‘easy’ (‘quite difficult’ for LVPEG-1) and tolerability as ‘okay’ (‘good’ for the control). In Part 2, per evening/morning intake, most patients rated tolerability as ‘very good’, ‘good’ or ‘acceptable’ and occurrence of symptoms as ‘none’. The taste of the solution was rated 44.9–57.0 on a visual analog scale of 0–100. Overall, most patients rated ease of drinking as ‘easy’ and tolerability as ‘acceptable’ or ‘good’.

## Discussion

PEG-based bowel preparations provide safe, high-quality cleansing but are not always well tolerated due to the high required fluid volume intake [15]. This study thus identified a novel low-volume PEG and Asc-based bowel preparation for further clinical development.

Five formulations with different combinations of low preparation volume (1–1.25L) and total required fluid volume (2–3L) were tested, all having an increased osmotic load and containing a high amount of ascorbate.

For ethical reasons cumulative stool output was the non-invasive pharmacodynamic endpoint in healthy volunteers in Part 1. For clinical proof of concept assessment, Part 2 was performed in patients undergoing screening colonoscopy. Stool output was utilized to confirm a repeatable experimental setting, given the difference in study population (older or at increased risk for CRC). A stable setting was also shown by the consistent performance of LVPEG-3 and the control in both study parts.

The clinical potential of all LVPEGs was demonstrated by triggering stool output peaks similar to those of 2L PEG + Asc (Fig. 2).

In Part 1, LVPEG-3 had the lowest preparation volume (1L) but achieved the highest mean 24-h-stool weight (3399 g) (Additional file 2: Table S2, Fig. 3). Among subjects taking LVPEG-3, 90% also reached clear effluent. LVPEG-1 failed to exceed the target stool weight of 2750 g, and LVPEG-2, whilst exceeding the target stool weight, showed no advantage of reversing the order of dosing, thus LVPEG-3 was selected as the lead for Part 2. 1L LVPEG-3 variants, LVPEG-4 (total required fluid volume of only 2L) and LVPEG-5 (reduced Asc level in the morning dose), were developed for parallel assessment in Part 2.

In Part 2, LVPEG-4 achieved the numerically highest mean 24-h-stool weight at 3215 g (Additional file 2: Table S2) and 80% of patients taking LVPEG-4 reached clear effluent. LVPEG-3 achieved 3050 g and 83.3% of patients in this group reached clear effluent. LVPEG-5 and the control both failed to reach the target stool weight of 2750 g. Clear effluent was also reached by fewer patients in these two groups.

LVPEG-3 and LVPEG-4 demonstrated 100% overall bowel cleansing success (90% CI of 0.88–1.00 vs the control for both). Both also achieved mean HCS segmental cleansing scores above 3: an indicator of high quality cleansing (Additional file 4: Table S3). Unlike LVPEG-3, however, LVPEG-4 achieved its high stool weight output performance level at a total required fluid volume consumption of only 2L (Additional file 3: Figure S1B). Total fluid volumes starting from 2 L were also sufficient for achieving clinical high-quality colon cleansing as measured with the HCS (Fig. 4c). LVPEG-4 was selected as the lead candidate for further development due to its high efficacy at a low volume. Among patients taking LVPEG-4, 29/30 achieved cumulative segmental scores of HCS 15 or higher, seemingly independent of their total fluid volume intake beyond the total required 2 L. The control showed larger cleansing variability. LVPEG-4 has thus demonstrated a clinically useful level of bowel cleansing efficacy as a qualitative clinical efficacy proof of concept.

Adverse events were few, gastrointestinal in nature and similar between groups. In both study parts 47–63% subjects taking LVPEGs experienced AEs (Table 3). Given the 37–50% range observed for 2 L PEG + Asc, the 50% rate for LVPEG-4 was not considered clinically relevant. The 2.5-fold increase of Asc per dose versus 2 L PEG + Asc and placing it all in a single (morning) dose did not increase total systemic exposure to Asc in patients receiving LVPEG-4 compared to those receiving the control. This is in line with previous observations that large doses of oral Asc are well tolerated [12, 16]. A small proportion of the Asc components and their metabolite, oxalic acid, were excreted through the urine in amounts comparable to the control group, hence posing no clinical risk to patients taking the bowel preparations. Electrolytes, which are naturally absorbed to maintain homeostasis during bowel preparation, were absorbed and excreted in nearly balanced proportions such that any resulting electrolyte shifts were not of clinical significance. Overall, these findings indicate that LVPEG-4 was considered tolerable and safe for further clinical investigation.

This study was powered to demonstrate mean stool weights larger than 2750 g in Part 1 only. The proof of concept demonstration of a potentially useful clinical efficacy and safety of the selected candidates was only qualitative. This study was not powered for quantitative comparisons between treatment groups or versus control. Patients took their preparations in clinic under the supervision of the investigator; the high compliance rates recorded in this study may be lower if patients self-administer at home.

## Conclusion

This study has confirmed that PEG and Asc (vitamin C), when combined in two carefully selected doses for split-dosing administration, can create a new, low-volume PEG-based bowel preparation with a clinically useful bowel-cleansing efficacy plus a promising safety and tolerability profile. The selected candidate 1 L PEG + Asc bowel preparation identified in this study, LVPEG-4, has now been investigated as part of a larger, multi-center, Phase 3 program with a more representative patient population in studies that are sufficiently powered to assess cleansing performance [17–19].

## Additional files

Additional file 1: **Table S1** Exclusion Criteria (DOCX 17 kb)

Additional file 2: **Table S2** Stool Weight (g). From Start of Dosing for 24 Hours, Parts 1 and 2 (Full Analysis Set) (DOCX 18 kb)

Additional file 3: **Figure S1**. Mean Cumulative 24-Hour Total Stool Weights. Mean Stool Weights (g) with 90% CI (g). **A)** Mean 24-h Stool Weights vs Preparation Volume. **B)** Mean 24-h Stool Weights vs Total Required Fluid Volume (Preparation Volume + Required Additional Clear Fluid Volume). Volumes in mL. Black circles indicate Part 1 of the study, white circles indicate Part 2. The horizontal grey line indicates the 2750 g mean stool weight target that was to be exceeded. (ZIP 621 kb)

Additional file 4: **Table S3.** Clinical Bowel Cleansing Efficacy. Harefield Cleansing Scale (HCS) Segmental Scores and Grades^a^ in Part 2. (DOCX 16 kb)

Additional file 5: **Table S4.** Pharmacokinetics. Plasma Ascorbic Acid PK Parameters in Part 2 (DOCX 16 kb)

Additional file 6: **Table S5.** Pharmacokinetics. Urine PK parameters (Sensitivity Analysis^a^) in Part 2 (DOCX 15 kb)

Additional file 7: **Table S6.** Pharmacokinetics. Feces PK Parameters (Sensitivity Analysis^a^) in Part 2 (DOCX 16 kb)

## Abbreviations

AE: Adverse event
Asc: Ascorbate
CRC: Colorectal cancer
FAS: Full analysis set
GI: Gastrointestinal
HCS: Harefield Cleansing Scale
LVPEG: Low-volume PEG + Asc
PEG: Polyethylene glycol
PK: Pharmacokinetic
SAF: Safety population
SD: Standard deviation
TEAE: Treatment-emergent adverse event
