# Supplementary material for: Novel 1 L polyethylene glycol-based bowel preparation (NER1006): proof of concept assessment versus standard 2 L polyethylene glycol with ascorbate – a randomized, parallel group, phase 2, colonoscopist-blinded trial
Source: BMC Gastroenterol. 2019 May 30;19:79. doi: 10.1186/s12876-019-0988-y (PMC6543558; doi:10.1186/s12876-019-0988-y)
Supplement: Supplementary file 1 — Table S1 Exclusion Criteria (DOCX 17 kb) [file 12876_2019_988_MOESM1_ESM.docx]

**Table S1: Exclusion Criteria**

| **Exclusion criterion** | **Description** |
| --- | --- |
| 1 | Part A only: subjects undergoing screening colonoscopy. |
| 2 | Presence of current clinically significant functional GI disorder (e.g., gastric emptying disorder, chronic constipation, irritable bowel syndrome [IBS]). |
| 3 | Regular use of laxatives or colon motility altering drugs in the last month. |
| 4 | Donation or loss of 500 mL or more of blood within 8 weeks prior to the first dose of investigational drug. |
| 5 | Any history or current presence of ileus, GI obstruction or perforation, GI tract cancer, inflammatory bowel disease (IBD) or colonic resection. |
| 6 | Known glucose-6-phosphatase dehydrogenase deficiency. |
| 7 | Known phenylketonuria. |
| 8 | History or evidence of any clinically significant cardiovascular or neurological disease, cardiac, renal or hepatic insufficiency. |
| 9 | Known hypersensitivity to polyethylene glycols and/or ascorbic acid. |
| 10 | History or evidence of any clinically relevant electrocardiogram (ECG) abnormalities and/or uncontrolled hypertension. |
| 11 | Evidence of dehydration. |
| 12 | Any evidence for clinically significant abnormal sodium or potassium levels or other clinically significant plasma electrolyte disturbances. |
| 13 | Females who are not postmenopausal with a positive pregnancy test. Females not using reliable methods of birth control if not postmenopausal. |
| 14 | Clinically relevant findings on physical examination based on the Investigator’s judgment. |
| 15 | Clinically relevant deviations of laboratory parameters from reference ranges at screening or check-in evaluation. |
| 16 | Positive serology for chronic viral hepatitis or human immunodeficiency virus (HIV) at screening. |
| 17 | History of drug or alcohol abuse within the 12 months prior to dosing or evidence of such abuse as indicated by the laboratory assays conducted during the screening or check-in evaluations. |
| 18 | Subjects who were unwilling to comply with the provisions of the study protocol. |
| 19 | Concurrent participation in an investigational drug study or participation within 3 months of study entry. |
| 20 | Subject had a condition or was in a situation, which in the Investigator’s opinion may have put the subject at significant risk, may have confounded the study results, or may have interfered significantly. |
| 21 | Previous participation in the study. |
| 22 | Persons who were ordered to live in an institution on court or authority order. |
