# Supplementary material for: Novel 1 L polyethylene glycol-based bowel preparation (NER1006): proof of concept assessment versus standard 2 L polyethylene glycol with ascorbate – a randomized, parallel group, phase 2, colonoscopist-blinded trial
Source: BMC Gastroenterol. 2019 May 30;19:79. doi: 10.1186/s12876-019-0988-y (PMC6543558; doi:10.1186/s12876-019-0988-y)
Supplement: Supplementary file 2 — Table S2 Stool Weight (g). From Start of Dosing for 24 Hours, Parts 1 and 2 (Full Analysis Set) (DOCX 18 kb) [file 12876_2019_988_MOESM2_ESM.docx]

**Table S2 Stool Weight (g).** From Start of Dosing for 24 Hours, Parts 1 and 2 (Full Analysis Set)

|  | **LVPEG-1** | **LVPEG-2** | **LVPEG-3** | **Control** | **LVPEG-3** | **LVPEG-4** | **LVPEG-5** | **Control** |
| --- | --- | --- | --- | --- | --- | --- | --- | --- |
| Study part | 1 | 1 | 1 | 1 | 2 | 2 | 2 | 2 |
| Patients, n | N=30 | N=29* | N=30 | N=30 | N=30 | N=30 | N=30 | N=30 |
| n | 30 | 29 | 30 | 29 | 30 | 30 | 30 | 30 |
| Mean | 2951.04 | 3218.69 | 3399.26 | 2490.77 | 3050.45 | 3215.08 | 2675.31 | 2487.14 |
| Median | 2978.75 | 3326.20 | 3513.00 | 2597.40 | 3065.70 | 3290.20 | 2657.95 | 2636.40 |
| SD | 873.767 | 809.888 | 575.778 | 879.242 | 705.406 | 634.254 | 612.154 | 634.689 |
| Minimum | 729.2 | 912.0 | 2114.5 | 375.8 | 1357.9 | 1501.7 | 1153.8 | 968.9 |
| Maximum | 4328.5 | 4559.1 | 4216.9 | 4505.0 | 4102.0 | 4433.3 | 3846.8 | 3430.8 |
| 90% Confidence Interval Around Mean | [2679.99; 3222.10] | [2962.85; 3474.52] | [3220.65; 3577.88] | [2213.02; 2768.51] | [2831.62; 3269.28] | [3018.32; 3411.84] | [2485.41; 2865.21] | [2290.25; 2684.03] |
| One sample t test (H_0_: stool weight output not more than 2750 g) | 0.2176 | 0.0042 | <0.0001 | 0.8764 | 0.0268 | 0.0004 | 0.4907 | 0.9691 |
| *One subject excluded from analysis as no stool collection data | | | | | | | | |
