# Supplementary figures and images for: Novel 1 L polyethylene glycol-based bowel preparation (NER1006): proof of concept assessment versus standard 2 L polyethylene glycol with ascorbate – a randomized, parallel group, phase 2, colonoscopist-blinded trial
Source: BMC Gastroenterol. 2019 May 30;19:79. doi: 10.1186/s12876-019-0988-y (PMC6543558; doi:10.1186/s12876-019-0988-y)

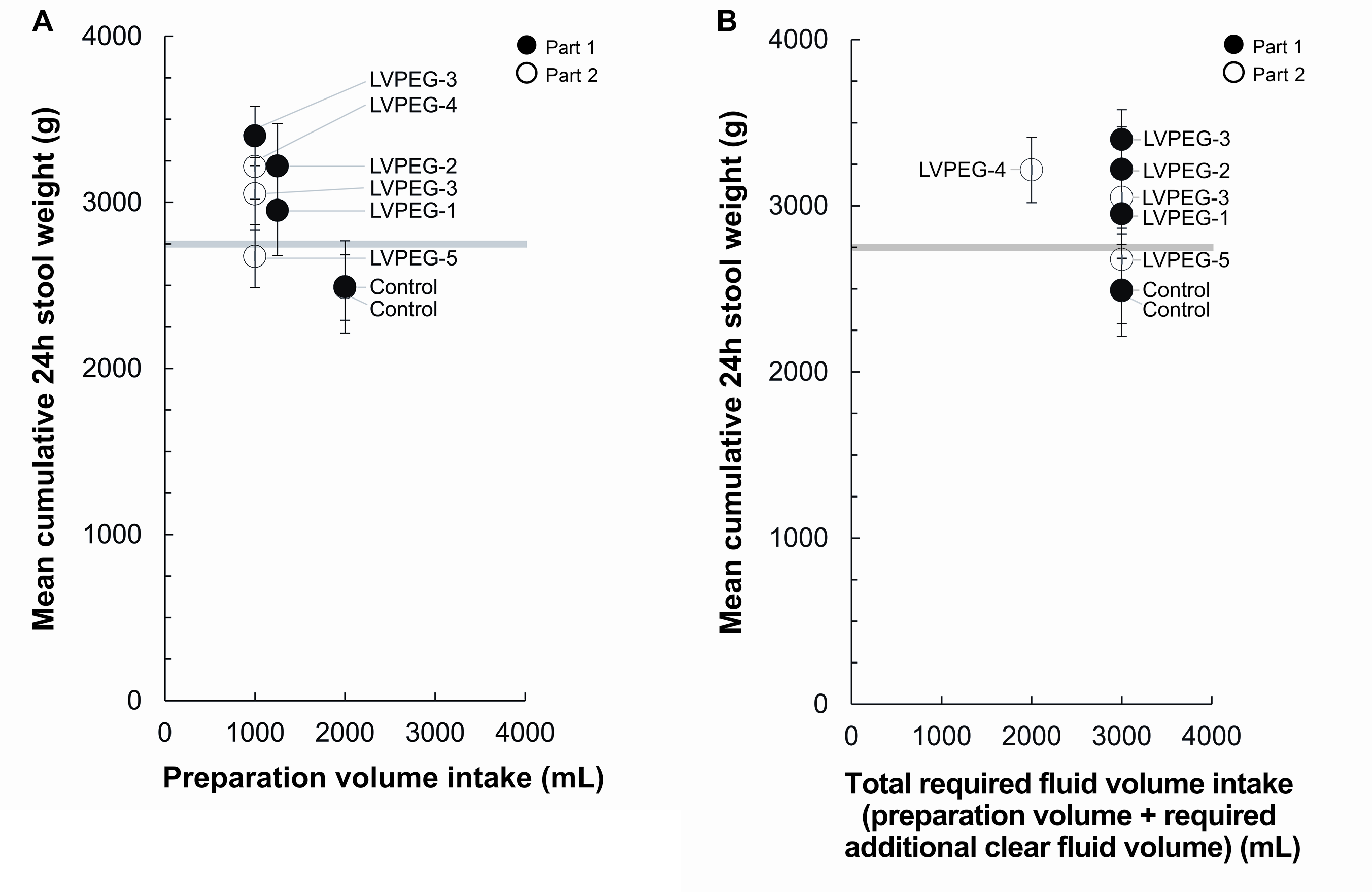

Supplement: Supplementary file 3 — Figure S1. Mean Cumulative 24-Hour Total Stool Weights. Mean Stool Weights (g) with 90% CI (g). A) Mean 24-h Stool Weights vs Preparation Volume. B) Mean 24-h Stool Weights vs Total Required Fluid Volume (Preparation Volume + Required Additional Clear Fluid Volume). Volumes in mL. Black circles indicate Part 1 of the study, white circles indicate Part 2. The horizontal grey line indicates the 2750 g mean stool weight target that was to be exceeded. (ZIP 621 kb) [file 12876_2019_988_MOESM3_ESM.zip › Supplementary figure 1R2.TIF]
