# Supplementary material for: Novel 1 L polyethylene glycol-based bowel preparation (NER1006): proof of concept assessment versus standard 2 L polyethylene glycol with ascorbate – a randomized, parallel group, phase 2, colonoscopist-blinded trial
Source: BMC Gastroenterol. 2019 May 30;19:79. doi: 10.1186/s12876-019-0988-y (PMC6543558; doi:10.1186/s12876-019-0988-y)
Supplement: Supplementary file 4 — Table S3. Clinical Bowel Cleansing Efficacy. Harefield Cleansing Scale (HCS) Segmental Scores and Gradesa in Part 2. (DOCX 16 kb) [file 12876_2019_988_MOESM4_ESM.docx]

**Table S3. Clinical Bowel Cleansing Efficacy.** Harefield Cleansing Scale (HCS) Segmental Scores and Grades^a^ in Part 2

|  |  | **LVPEG-3** | **LVPEG-4** | **LVPEG-5** | **Control** |
| --- | --- | --- | --- | --- | --- |
| Patients, n |  | 30 | 30 | 30 | 30 |
| Segmental Score (mean ± SD) | |  |  |  |  |
|  | Ascending Colon plus Cecum | 3.2 (0.7) | 3.2 (0.4) | 3.0 (0.8) | 2.6 (1.0) |
|  | Transverse Colon | 3.2 (0.7) | 3.4 (0.5) | 3.1 (0.6) | 3.0 (0.8) |
|  | Descending Colon | 3.3 (0.6) | 3.6 (0.5) | 3.2 (0.7) | 2.6 (0.6) |
|  | Sigmoid Colon | 3.2 (0.7) | 3.4 (0.6) | 2.9 (0.8) | 2.6 (0.6) |
|  | Rectum | 3.1 (0.7) | 3.2 (0.4) | 2.9 (0.8) | 2.6 (1.0) |
| HCS Grade, n (%) | |  |  |  |  |
|  | A | 22 (73.3) | 28 (93.3) | 20 (66.7) | 6 (20.0) |
|  | B | 8 (26.7) | 2 (6.7) | 7 (23.3) | 21 (70.0) |
|  | C | 0 (0) | 0 (0) | 3 (10.0) | 2 (6.7) |
|  | D | 0 (0) | 0 (0) | 0 (0) | 1 (3.3) |
| SD, standard deviation;  ^a^HCS, Harefield Cleansing Scale. According to the HCS: 0 = irremovable, heavy, hard stools; 1 = semi-solid, only partially removable stools; 2 = brown liquid/removable semi-solid stools; 3 = clear liquid; 4 = empty and clean; A = all five segments scored 3 or 4; B = one or more segments scored 2, remaining segments scored 3 or 4; C = one or more segments scored 1, remaining segments scored 2, 3 or 4; D = one or more segments scored 0. | | | | | |
