# Supplementary material for: Novel 1 L polyethylene glycol-based bowel preparation (NER1006): proof of concept assessment versus standard 2 L polyethylene glycol with ascorbate – a randomized, parallel group, phase 2, colonoscopist-blinded trial
Source: BMC Gastroenterol. 2019 May 30;19:79. doi: 10.1186/s12876-019-0988-y (PMC6543558; doi:10.1186/s12876-019-0988-y)
Supplement: Supplementary file 5 — Table S4. Pharmacokinetics. Plasma Ascorbic Acid PK Parameters in Part 2 (DOCX 16 kb) [file 12876_2019_988_MOESM5_ESM.docx]

**Table S4. Pharmacokinetics.** Plasma Ascorbic Acid PK Parameters in Part 2

|  | **Visit** | **Statistic** | **LVPEG-3** | **LVPEG-4** | **LVPEG-5** | **Control** |
| --- | --- | --- | --- | --- | --- | --- |
| Patients, n |  |  | 29 | 30 | 30 | 30 |
| AUC_0‑t_, h*μg/ml |  |  |  |  |  |  |
|  | DAY 1 | n | 9 | 14 | 13 | 30 |
|  |  | Mean | 11.1 | 7.2 | 4.8 | 160.1 |
|  |  | SD | 12.5 | 7.4 | 6.1 | 38.8 |
|  | DAY 2 | n | 29 | 30 | 30 | 30 |
|  |  | Mean | 398.5 | 378.9 | 246.9 | 133.4 |
|  |  | SD | 116.2 | 139.2 | 63 | 41.3 |
| C_max_, μg/ml |  |  |  |  |  |  |
|  | DAY 1 | n | 9 | 14 | 13 | 30 |
|  |  | Mean | 2.9 | 3 | 1.8 | 42.9 |
|  |  | SD | 2.5 | 2.6 | 1.5 | 11.9 |
|  | DAY 2 | n | 29 | 30 | 30 | 30 |
|  |  | Mean | 88.1 | 81.1 | 52.3 | 31.5 |
|  |  | SD | 28.2 | 28.3 | 14.3 | 8.9 |
| t_max_, h |  |  |  |  |  |  |
|  | DAY 1 | n | 9 | 14 | 13 | 30 |
|  |  | Mean | 3.5 | 2.5 | 3.3 | 3.2 |
|  |  | SD | 2.1 | 1.4 | 1.2 | 0.4 |
|  | DAY 2 | n | 29 | 30 | 30 | 30 |
|  |  | Mean | 3.2 | 3.2 | 3.1 | 3 |
|  |  | SD | 0.9 | 0.7 | 0.4 | 0.5 |
| AUC_0-t_, area under the concentration curve from time zero to the last quantifiable concentration; SD, standard deviation; C_max_, maximum observed concentration; t_max_, time to occurrence of Cmax. | | | | | | |
