# Supplementary material for: Novel 1 L polyethylene glycol-based bowel preparation (NER1006): proof of concept assessment versus standard 2 L polyethylene glycol with ascorbate – a randomized, parallel group, phase 2, colonoscopist-blinded trial
Source: BMC Gastroenterol. 2019 May 30;19:79. doi: 10.1186/s12876-019-0988-y (PMC6543558; doi:10.1186/s12876-019-0988-y)
Supplement: Supplementary file 6 — Table S5. Pharmacokinetics. Urine PK parameters (Sensitivity Analysisa) in Part 2 (DOCX 15 kb) [file 12876_2019_988_MOESM6_ESM.docx]

**Table S5. Pharmacokinetics.** Urine PK parameters (Sensitivity Analysis^a^) in Part 2

|  | **Collection Interval** | **Statistic** | **LVPEG-3** | **LVPEG-4** | **LVPEG-5** | **Control** |
| --- | --- | --- | --- | --- | --- | --- |
| Patients, n |  |  | 29 | 30 | 30 | 30 |
| Ascorbic Acid, Amount Excreted (mg) | 0–12h | n | 29 | 30 | 30 | 30 |
|  |  | Mean | 1.2 | 1 | 2.2 | 321.1 |
|  |  | SD | 1.2 | 1.2 | 2.4 | 227.2 |
|  | 12–24h | n | 29 | 30 | 30 | 30 |
|  |  | Mean | 485.1 | 458.9 | 317.7 | 687.2 |
|  |  | SD | 377.9 | 361.5 | 251.7 | 404.1 |
|  | 0–24h | n | 29 | 30 | 30 | 30 |
|  |  | Mean | 486.3 | 459.9 | 319.8 | 1008.4 |
|  |  | SD | 378.1 | 361.5 | 251.6 | 445.3 |
| Oxalic Acid, Amount Excreted (mg) | 0–12h | n | 29 | 30 | 30 | 30 |
|  |  | Mean | 1.9 | 1.4 | 1.8 | 1.9 |
|  |  | SD | 1.5 | 1.5 | 1.8 | 1.6 |
|  | 12–24h | n | 29 | 30 | 30 | 30 |
|  |  | Mean | 3.9 | 4.2 | 4.9 | 4.9 |
|  |  | SD | 2.7 | 2.7 | 2.8 | 3.6 |
|  | 0–24h | n | 29 | 30 | 30 | 30 |
|  |  | Mean | 5.8 | 5.6 | 6.7 | 6.8 |
|  |  | SD | 3.5 | 3.1 | 3.5 | 4.2 |
| SD*,* standard deviation ^a^The sensitivity analysis was performed without outliers. Outliers were identified as any value more than 1.5 interquartile ranges below the first quartile or above the third quartile. | | | | | | |
