# Supplementary material for: Novel 1 L polyethylene glycol-based bowel preparation (NER1006): proof of concept assessment versus standard 2 L polyethylene glycol with ascorbate – a randomized, parallel group, phase 2, colonoscopist-blinded trial
Source: BMC Gastroenterol. 2019 May 30;19:79. doi: 10.1186/s12876-019-0988-y (PMC6543558; doi:10.1186/s12876-019-0988-y)
Supplement: Supplementary file 7 — Table S6. Pharmacokinetics. Feces PK Parameters (Sensitivity Analysisa) in Part 2 (DOCX 16 kb) [file 12876_2019_988_MOESM7_ESM.docx]

**Table S6. Pharmacokinetics.** Feces PK Parameters (Sensitivity Analysis^a^) in Part 2

|  | **Collection Interval** | **Statistic** | **LVPEG-3** | **LVPEG-4** | **LVPEG-5** | **Control** |
| --- | --- | --- | --- | --- | --- | --- |
| Patients, n |  |  | 29 | 30 | 30 | 30 |
| Ascorbic Acid, Amount Excreted (g) | 0–12h | n | 29 | 30 | 30 | 30 |
|  |  | Mean | 0.022 | 0.021 | 0.020 | 3.301 |
|  |  | SD | 0.010 | 0.008 | 0.010 | 2.061 |
|  | 12–24h | n | 29 | 30 | 30 | 30 |
|  |  | Mean | 30.832 | 33.886 | 22.417 | 3.740 |
|  |  | SD | 9.172 | 10.174 | 6.034 | 2.664 |
|  | 0–24h | n | 29 | 30 | 30 | 30 |
|  |  | Mean | 30.854 | 33.907 | 22.438 | 7.041 |
|  |  | SD | 9.177 | 10.177 | 6.038 | 4.457 |
| Oxalic Acid, Amount Excreted (g) | 0–12h | n | 29 | 30 | 28 | 30 |
|  |  | Mean | 0.150 | 0.081 | 0.085 | 0.117 |
|  |  | SD | 0.114 | 0.061 | 0.042 | 0.080 |
|  | 12–24h | n | 29 | 30 | 28 | 30 |
|  |  | Mean | 0.089 | 0.107 | 0.079 | 0.069 |
|  |  | SD | 0.034 | 0.032 | 0.023 | 0.021 |
|  | 0–24h | n | 29 | 30 | 28 | 30 |
|  |  | Mean | 0.238 | 0.188 | 0.164 | 0.186 |
|  |  | SD | 0.119 | 0.066 | 0.050 | 0.090 |
| PEG3350, Amount Excreted (g) | 0–12h | n | 28 | 30 | 30 | 30 |
|  |  | Mean | 65.997 | 58.736 | 57.263 | 51.949 |
|  |  | SD | 23.801 | 23.360 | 28.641 | 26.180 |
|  | 12–24h | n | 28 | 30 | 30 | 30 |
|  |  | Mean | 53.238 | 62.855 | 64.388 | 99.656 |
|  |  | SD | 15.286 | 21.501 | 17.632 | 38.619 |
|  | 0–24h | n | 28 | 30 | 30 | 30 |
|  |  | Mean | 119.235 | 121.591 | 121.650 | 151.605 |
|  |  | SD | 24.619 | 28.736 | 24.064 | 49.404 |
| SD*,* standard deviation ^a^The sensitivity analysis was performed without outliers. Outliers were identified as any value more than 1.5 interquartile ranges below the first quartile or above the third quartile. | | | | | | |
